# Comparison of two ^18^F-fluorinated glycopeptides for PET imaging of the functional liver mass

**DOI:** 10.1186/s41181-026-00445-z

**Published:** 2026-04-04

**Authors:** Maximilian A. Zierke, Katharina Hofer, Kimia Samadikhah, Christine Rangger, Carmen Wängler, Björn Wängler, Anna Junker, Andreas M. Schmid, Roland Haubner

**Affiliations:** 1 Department of Nuclear Medicine, Medical University Innsbruck, Anichstr. 35, 6020 Innsbruck, Austria; 2 Werner Siemens Imaging Center, Department of Preclinical Imaging and Radiopharmacy, Eberhard Karls University Tübingen, Röntgenweg 13, 73076 Tübingen, Germany; 3 Biomedical Chemistry, Clinic of Radiology and Nuclear Medicine, Medical Faculty Mannheim, Heidelberg University, Theodor-Kutzer-Ufer 1-3, 68167 Mannheim, Germany; 4 Research Campus M²OLIE, Medical Faculty Mannheim, Heidelberg University, 68167 Mannheim, Germany; 5 Molecular Imaging and Radiochemistry, Clinic of Radiology and Nuclear Medicine, Medical Faculty Mannheim, Heidelberg University, Theodor-Kutzer-Ufer 1-3, 68167 Mannheim, Germany; 6 Cluster of Excellence iFIT (EXC 2180) “Image-Guided and Functionally Instructed Tumor Therapies”, University of Tübingen, 73076 Tübingen, Germany

**Keywords:** Asialoglycoprotein receptor, Positron emission tomography, Fluorine-18, Silicon-fluoride acceptor, Aluminum fluoride

## Abstract

**Background:**

The non-invasive determination of the asialoglycoprotein receptor (ASGR) expression on liver tissue might be a predictive parameter helping to prevent severe liver diseases as well as liver failure after surgery and transplantation. Recently, we introduced [^68^Ga]Ga-NODAGA-NonaLysan, which showed even a higher liver uptake as the gold standard [^99m^Tc]Tc-Galactosyl Serum Albumin. Here we describe the synthesis and evaluation of two ^18^F-labeled analogues, prepared using either a SiFA- or an AlF-labeling approach.

**Results:**

The two precursors could be produced in high purity (> 97%). Both labeling strategies allowed production of the radiopharmaceuticals in high radiochemical purity. The radiochemical yield was up to 58% d.c. for [^18^F]**SiFA-NonaLysan** and up to 71% d.c. for Al[^18^F]F-**NOTA-6-Ahx-NonaLysan**. In vitro evaluation showed high stability in PBS and human serum. Both compounds possessed nanomolar affinity for the ASGR (IC_50_ = 0.9 ± 1.1 nM and 3.0 ± 2.1 nM, respectively). However, based on the design differences, Al[^18^F]F-**NOTA-6-Ahx-NonaLysan** showed a 100-fold lower *logD* as found for [^18^F]**SiFA-NonaLysan**. In consequence, the protein binding effect was higher for [^18^F]**SiFA-NonaLysan**, and the lipophilic character drastically affected the pharmacokinetic pattern. Initial liver uptake was higher for [^18^F]**SiFA-NonaLysan** (100% ID/g vs. 70% ID/g 10 min p.i.), but was accompanied by a quick organ washout and activity accumulation in the intestines. A much better activity retention was observed for Al[^18^F]F-**NOTA-6-Ahx-NonaLysan**. PET/MR imaging confirmed the differences in liver uptake, with a higher retention found for the latter. Based on the receptor function, both compounds are internalized and subsequently degraded. For Al[^18^F]F-**NOTA-6-Ahx-NonaLysan**, all radioactive liver metabolites found were more hydrophilic than the intact compound. For [^18^F]**SiFA-NonaLysan**, the contrary is the case, and almost all liver metabolites were more lipophilic. The hepatobiliary excretion of these metabolites prevents a stable activity retention in the hepatic tissue.

**Conclusion:**

In this study, we successfully synthesized two new ^18^F-labeled radiopharmaceuticals targeting the ASGR. The in vivo evaluation revealed two different pharmacokinetic profiles. Extremely high uptake was found for [^18^F]**SiFA-NonaLysan** in the initial phase, followed by a quick organ washout. Al[^18^F]F-**NOTA-6-Ahx-NonaLysan** showed a lower but more stable activity retention in the liver, indicating advantageous imaging properties.

**Supplementary Information:**

The online version contains supplementary material available at 10.1186/s41181-026-00445-z.

## Background

According to data from the World Health Organization (WHO) regarding the leading causes of death, liver diseases rank in 11th position with more than 2 million deaths per year. Among those, the most apparent liver diseases are cirrhosis, viral hepatitis, and liver cancer (Devarbhavi et al. 2023). Examples of preventive measures for such diseases include reducing alcohol consumption, vaccinating against hepatitis viruses, and, last but not least, the availability of non-invasive imaging techniques that enable early detection of cirrhotic nodules. One such imaging technique is based on [^99m^Tc]Tc-Galactosyl Serum Albumin ([^99m^Tc]Tc-GSA), which has shown great value for the estimation of the hepatic reserve in patients suffering from fibrosis (Iguchi et al. 2003; Kozaki et al. 2024), cirrhosis (Sasaki et al. 1999), hepatocellular carcinoma (Kaibori et al. 2011), and acute and chronic liver disease (Kudo et al. 1993). In addition, it has become an important tool for surgery planning and living-donor transplantation due to its high predictive value for post-hepatectomy liver failure in clinical routine (Kokudo et al. 2003; Ohno et al. 2004; Iida et al. 2015; Nakamura et al. 2018; Suzuki et al. 2021). The tracer targets the asialoglycoprotein receptor (ASGR), a receptor expressed at high levels and almost exclusively on functional hepatocytes. In liver disease, ASGR expression is decreased, enabling non-invasive diagnosis of pathologic processes (Sawamura et al. 1985; Shao et al. 2019). Unfortunately, this compound is only clinically approved in Japan, which has led to the development of novel ASGR-targeting tracers. Currently, one of the most promising alternatives is a radiopharmaceutical named *Dolacga* from Taiwan. It is a ^68^Ga-labeled hexavalent lactoside for PET imaging of liver function (Yu et al. 2018). In addition to this, we have recently developed [^68^Ga]Ga-NODAGA-NonaLysan, a peptidic galactose nonamer, which was selected from a small set of glycopeptides. This promising compound reached even higher liver uptake in healthy mice than the already clinically used [^99m^Tc]Tc-GSA (Zierke et al. 2025).

However, for PET imaging worldwide not gallium-68 but fluorine-18 is the most widely used radionuclide (Rong et al. 2023). Its half-life of 110 min exceeds that of gallium-68 (*t*_*1/2*_ = 67.9 min) and hence, allows for longer production processes and even shipping of radiopharmaceuticals to distant PET centers (Notni et al. 2019). In addition, it comes with a low positron energy (E_av β+_ = 250 keV, E_max β+_ = 634 keV), resulting in PET images with higher resolution compared to gallium-68 (E_av β+_ = 862 keV, E_max β+_ = 1899 keV) (Notni et al. 2019). In the past, generations of scientists have focused on strategies to incorporate ^18^F-labels into organic molecules and peptides. Most of the commonly used fluorination methods range from alkylic (S_N_2) and aromatic (S_N_Ar) substitutions to diaryliodonium salts and prosthetic groups (Schubiger et al. 2007; Cai et al. 2008). Alternatively, click chemistry-based approaches have also been reported (Iddon et al. 2011; Sun et al. 2020). The problem with these strategies is the need for multistep syntheses and requirements for HPLC purification steps. The discovery of novel radiofluorination techniques *via* conjugation and chelation, such as the SiFA-chemistry (Schirrmacher et al. 2006) and the aluminum fluoride formation (McBride et al. 2009), has tremendously facilitated the synthesis and evaluation of ^18^F-labeled radioligands. These conjugates allow radiofluorination in a one-pot manner followed by purification *via* solid-phase extraction. Several review articles report the successful application of these novel fluorination techniques (Wängler et al. 2012; Archibald et al. 2021). Some SiFA-conjugated radioligands have even found their way into clinical routine, as seen for SiFAlin-TATE (Lindner et al. 2020) and rh-PSMA 7.3 (Posluma^®^) (Rauscher et al. 2024). On the other hand, the Al[^18^F]F strategy works with chelator-based radioligands, hence allowing a direct comparison of the fluorinated compound to the ^68^Ga-labeled counterpart (Ahenkorah et al. 2023; Liu et al. 2024).

Building upon previous work with [^68^Ga]Ga-NODAGA-NonaLysan (Zierke et al. 2025), we sought to further improve the diagnostic value and clinical availability of our liver-targeting radiotracer. Specifically, our aim was to develop a fluorinated analogue without the need for a laborious synthetic route. In this study, we synthesized [^18^F]**SiFA-NonaLysan** and Al[^18^F]F-**NOTA-6-Ahx-NonaLysan**, and preclinically evaluated the two tracers regarding their suitability as PET agents for liver function imaging.

## Material & methods

Solvents, chemicals, and reagents for solid-phase peptide synthesis were purchased from Merck (Darmstadt, Germany) or VWR International GmbH (Wien, Austria). 1-Hydroxy-7-azabenzotriazol was supplied by Activate Scientific (Prien am Chiemsee, Germany) as a 1 M solution in dimethylacetamide. SiFA benzoic acid was prepared according to a published procedure (Iovkova et al. 2009). 1-Azido-1-deoxy-β-d-galactopyranoside tetraacetate and 1-azido-1-deoxy-β-d-galactopyranoside were obtained from Merck. NOTA-NHS was purchased from Chematech (Dijon, France). Human recombinant ASGR1 (#4394-AS) was obtained from R&D Systems (Minneapolis, USA). Human *α*1-glycoprotein was commercially available at Merck. [^18^F]Fluoride was produced at the Department of Nuclear Medicine of the Klinikum rechts der Isar, Munich, Germany (1.3–4.9 GBq/mL). It was also produced at the Werner Siemens Imaging Center, Tübingen, Germany (0.4–1.8 GBq/mL). A description of the equipment used for ^18^F-labeling in the PET imaging studies can be found in the Supplementary Information (SI).

Radio-thin layer chromatography was performed on Polygram SIL G/UV 254 (0.2 mm silicagel) stripes with a solvent mixture of water/acetonitrile (2:3) (vol/vol) supplemented with 10% 2 M NaOAc and 1% TFA (Wurzer et al. 2020). Readout of the stripes was accomplished using a LabLogic ScanRAM TLC-scanner (Broomhill, Sheffield, U.K.). Analytical HPLC was performed on a Thermo Scientific Ultimate 3000 HPLC (Waltham, Massachusetts, USA) equipped with a quaternary LPG-3400SD pump, WPS-3000SL autosampler, RS column compartment, and variable wavelength detector at a flow rate of 1 mL/min. For radio-HPLC applications, a GabiStar detector (Raytest, Straubenhardt, Germany) was added. All devices were controlled with the Chromeleon 7 software. The column in use was a Dr. Maisch ReproSil Pur C_18_ AQ, 5 μm, 120 Å, 150 × 4.6 mm. Semi-preparative HPLC was performed on a Thermo Scientific Ultimate 3000 HPLC equipped with a binary RS pump and variable wavelength detector at a flow rate of 8 mL/min. The column in use was a Dr. Maisch RepsoSil Pur C_18_ AQ, 5 μm, 120 Å, 250 × 20 m. Millipore water (Solvent A) and acetonitrile (Solvent B) both containing 0.1 vol % TFA served as eluents. Electrospray ionization mass spectrometry (ESI-MS) in positive ionization mode were recorded on a Shimadzu Nexera-2050 LC-MS (Kyoto, Japan) equipped with a SCL-40 system controller, DGU-405 degassing unit, LC-40D pump and SPD-40 V UV-VIS detector. LiChrosolv^®^ Water (Solvent A) and LiChrosolv^®^ hypergrade acetonitrile (Solvent B), both containing 0.1 vol-% LiChropur^®^ formic acid, served as eluents. Radioactive samples were measured in a HIDEX AMG automatic gamma counter (Turku, Finland). The procedures for stability assays in PBS and human blood serum, the determination of protein binding, and the *logD*-value followed established protocols (Hörmann et al. 2020). The binding affinity (IC_50_) towards the immobilized human recombinant ASGR1 was performed according to a previously published procedure with minor modifications (Zierke et al. 2024a, b). Synthesis of the labeling precursors **SiFA-NonaLysan** and **NOTA-6-Ahx-NonaLysan** including mass spectra and HPLC chromatograms is provided in the SI.

### [^18^F]SiFA-labeling

 [^18^F]Fluoride was immobilized on a strong anion exchange cartridge (Sep-Pak Accell Plus QMA Carbonate Plus Light, 46 mg, 40 μm; Waters). The cartridge was flushed with 10 mL of anhydrous acetonitrile and 20 mL of air to remove any residing solvent. For elution of the activity, a lyophilized kit containing 110 µmol Kryptofix 222 (Merck) and 100 µmol KOH (Merck) was freshly dissolved in 500 µL of anhydrous acetonitrile and passed through the column. The eluate was partially neutralized to a pH of 7–8 with 30 µmol (1 M, 30 µL) of oxalic acid (99.999% trace metal basis) in anhydrous acetonitrile. An aliquot of this solution (450–630 MBq) was mixed with a stock solution of precursor (20 nmol, 1 mM, 20 µL) in anhydrous DMSO (> 99.9%). The resulting mixture was incubated for 10 min at 70 °C at a shaker speed of 1,300 rpm. For work-up, the mixture was diluted with 10 mL of PBS/HCl (pH 3) and loaded onto a preconditioned tC_18_ Sep-Pak Plus Light cartridge (5 mL of ethanol, 10 mL of Millipore water, 1 mL of air). Unbound [^18^F]fluoride was removed by washing with 10 mL of PBS/HCl (pH 3). Other impurities were removed by application of a step gradient [5:95, 10:90, 20:80, and 30:70 ethanol/water (vol/vol) with a volume of 1 mL each]. The radioligand was eluted using 1 mL of ethanol/water (50:50) (vol/vol). The purity of the final product was analyzed by radio-HPLC and radio-TLC. For biodistribution experiments and PET/MR imaging studies the radioligand was diluted 1:5 with PBS.

### Al[^18^F]F-labeling

 [^18^F]Fluoride was immobilized on a strong anion exchange cartridge (Sep-Pak Accell Plus QMA Carbonate Plus Light, 46 mg, 40 μm; Waters), preconditioned with 5 mL metal-free 0.5 M NaOAc/HOAc buffer (pH 4), 10 mL Millipore water, and dried with 3 mL of air. The cartridge was flushed with 5 mL of Millipore water and the activity was eluted inversely using 1 mL of a 0.5 M NaOAc/HOAc buffer (pH 4) in 200 µL fractions. From the fraction with the highest activity concentration, an aliquot of 100 µL (520–840 MBq) was mixed with 30 nmol (2 mM, 15 µL) of AlCl_3_ (99.999% trace metal basis, Merck) in 0.1 M NaOAc/HOAc buffer (pH 4). This solution was diluted 1:1 with ethanol and allowed to rest at room temperature for 5 min. Upon the formation of Al[^18^F]F^2+^, 55 nmol (1 mM, 55 µL) of precursor in ethanol/water (50:50) (vol/vol) was added. The resulting mixture was incubated for 20 min at 100 °C at a shaker speed of 1,300 rpm. For work-up, the reaction solution was diluted with 10 mL of Millipore water and loaded onto a preconditioned C18 SepPak Light Plus cartridge (5 mL of ethanol, 10 mL of Millipore water, 1 mL of air). Unbound Al[^18^F]F^2+^ was removed by washing with 10 mL of Millipore water. The radioligand was eluted with 1 mL of ethanol/water (50:50) (vol/vol). The purity of the final product was analyzed by radio-HPLC and radio-TLC as stated above. For biodistribution experiments and PET/MR imaging studies, the radioligand was diluted 1:5 with PBS.

### Biodistribution studies

 Animal experiments were performed according to the Austrian animal experiments law (BGBl. I Nr. 114/2012) and the institution’s animal welfare standards as approved by the Austrian Federal Ministry of Education, Science and Research (BMBWF, 2022 − 0.311.708). For biodistribution studies, 6-week old healthy female BALB/c mice (*n* = 3, Charles River Laboratories, Sulzfeld, Germany) were injected with either 200 pmol [^18^F]**SiFA-NonaLysan** (A_m_ = 3–15 MBq/nmol) or 400 pmol Al[^18^F]F-**NOTA-6-Ahx-NonaLysan** (A_m_ = 3–5 MBq/nmol) and sacrificed by cervical dislocation after 10, 30–60 min post injection (p.i.). Mice were dissected, blood and organs were weighed and the radioactivity content was measured in a gamma counter. Uptake values are presented as injected dose percent per gram (ID %/g).

### PET/MR imaging studies

Imaging experiments were conducted according to approved procedures (R09/21G) and followed the same protocols as previous studies (Zierke et al. 2024a, b). In brief, animals were anesthetized, placed in the scanner, and injected with ~ 1 MBq of the corresponding tracer. After a dynamic PET measurement, animals were transferred to an MRI for anatomical reference. Images were co-registered, and regions of interest were analyzed. The corresponding hepatic accumulation indices LHL15 [activity in the liver/(activity in the liver + activity in the heart) determined 15 min p.i.] were derived from the individual time activity curves (TACs).

### Metabolite analysis

Healthy female BALB/c mice were injected with 5 MBq [^18^F]SiFA-NonaLysan or 2.5 MBq Al[^18^F]F-NOTA-6-Ahx-NonaLysan and euthanized after 10 (*n* = 2) or 60 min (*n* = 2) by cervical dislocation. Urine and blood samples were collected in 1.5 mL Eppendorf tubes. Urine was centrifuged (14,500 rcf, 5 min), and the supernatant was diluted 1:10 with Millipore water. Blood was allowed to coagulate, and cellular components were separated by centrifugation (14,500 rcf, 5 min). The supernatant containing the serum was removed and mixed 1:1 with acetonitrile for the precipitation of proteins. Precipitates were spun down (14,500 rcf, 5 min), and the supernatant was diluted 1:3 with water. Activity in pellets and supernatants was quantified in a gamma counter to determine extraction efficiency. Protein-free supernatants were analyzed by radio-HPLC. The liver and intestine were excised and transferred into 50 mL Falcon tubes, followed by the addition of PBS (1–3 mL). Organs were homogenized on ice for 1 min using an IKA Ultra-Turrax (Staufen, Germany) and pelleted (3,000 rcf, 10 min). The supernatants were removed and transferred into a 1.5 mL Eppendorf tube for another centrifugation step (14,500 rcf, 10 min). An aliquot (200 µL) of the supernatant was mixed 1:1 with acetonitrile and vortexed for protein precipitation. Precipitates were spun down (14,500 rcf, 10 min), and the protein-free supernatant was diluted 1:5 with Millipore water. Activity in pellets and supernatants was quantified in a gamma counter to determine extraction efficiency. Protein-free supernatants were analyzed by radio-HPLC using a Macherey-Nagel column (Nucleosil 120-5 C18 125 × 4.6 mm, 5 μm).

## Results

### Chemical synthesis

**Fig. 1:**
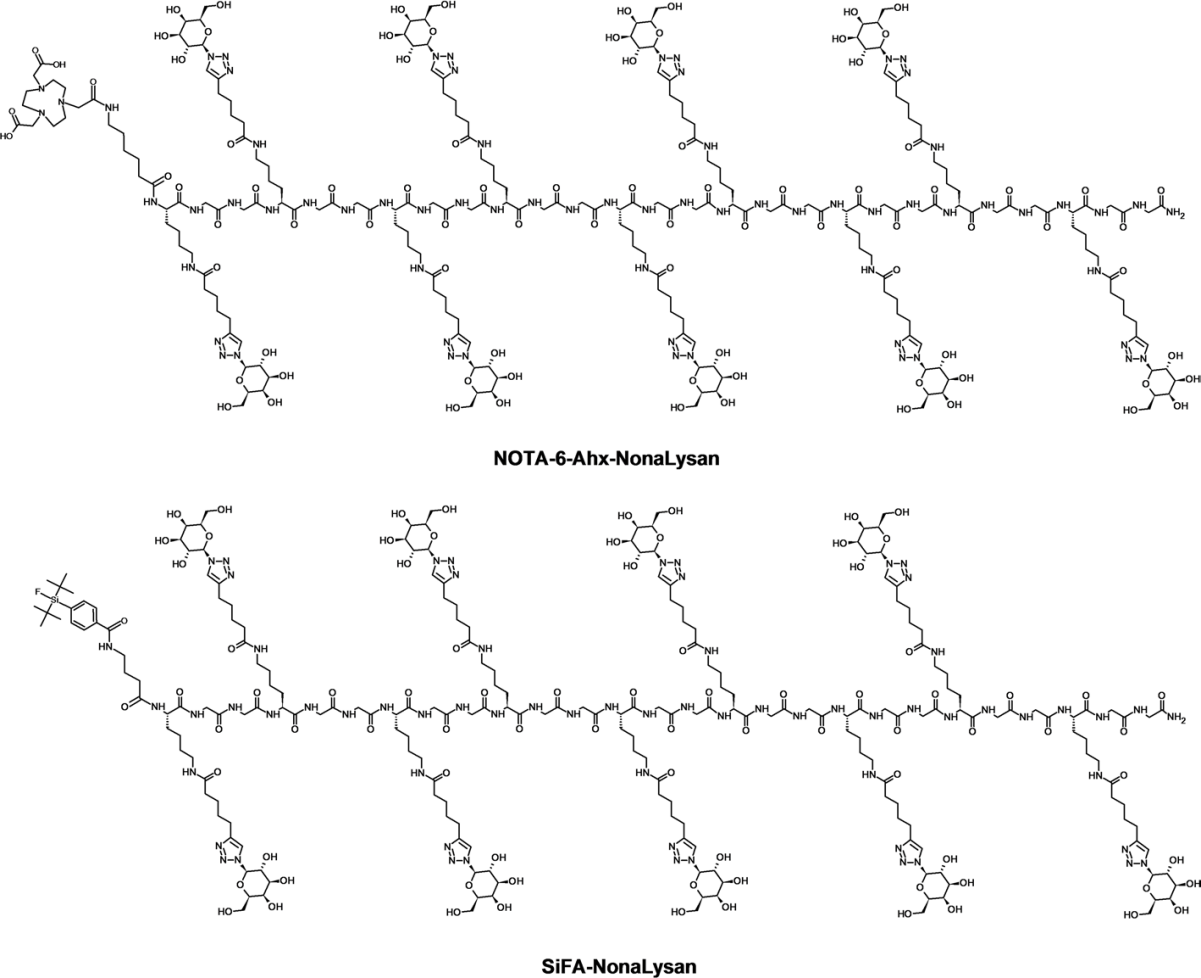
Chemical structures of **NOTA-6-Ahx-NonaLysan** & **SiFA-NonaLysan**

The preparation of the labeling precursors was achieved *via* a recently published fragment condensation approach (Fig. 1 and Fig. S1) (Zierke et al. 2025). Fmoc-K(6-heptynoic acid)GG-OH was immobilized on a Rink amide resin and served as the general building block. Peptides with 9 lysine residues were obtained by repetitive coupling cycles. In the case of **SiFA-NonaLysan**, γ-butyric acid was introduced as an *N*-terminal spacer, followed by coupling of SiFA-benzoic acid. After cleavage from the resin, the crude peptide was galactosylated and purified *via* semi-preparative HPLC. In the case of the NOTA-conjugated peptide, 6-amino-hexanoic acid was introduced as an *N*-terminal spacer, followed by cleavage from the solid support. The peptidic backbone was galactosylated in solution using copper catalyzed azide-alkyne cyclodaddition. After semi-preparative HPLC purification of the intermediate, NOTA-NHS ester was conjugated in anhydrous DMSO. **SiFA-NonaLysan** was obtained in a total yield of 2%, **NOTA-6-Ahx-NonaLysan** in yields of 3%, respectively (Table 1). Both compounds showed high purity (> 97%) when analyzed by analytical RP-HPLC *(see SI)*.

**Table 1:** MS data, HPLC retention time, purity and yields of the glycopeptides

	Monoisotopic Mass calcd. (Da)	Monoisotopic Mass found (m/z)	t_*R*_ HPLC^a^ (min)	Purity^b^ (%)	Yield^c^ (%)
**SiFA-NonaLysan**	5363.60	1788.27 [M + 3 H]^3+^	12.4	> 97	2
**NOTA-6-Ahx-NonaLysan**	5412.63	1806.27 [M + 3 H]^3+^	7.7	> 97	3

^*a*^ ReproSil Pur C_18_ AQ Gradient: 5–60% B in 15 min, 1 mL/min;

^*b*^ Determined by HPLC;

^*c*^ Calculated based on the initial amount of Fmoc-K(6-heptynoic acid)GG-OH immobilized on the Rink amide resin.

### Radiofluorination

^18^F-Labeling of **SiFA-NonaLysan** occurred *via* an isotopic exchange reaction according to a previously published procedure for SiFA-bearing ligands (Wurzer et al. 2020). [^18^F]Fluoride was fixed on a QMA cartridge and dried *via* the Munich Method (Wessmann et al. 2012). Elution of the activity from the cartridge was accomplished with a solution of [K^+^ _2.2.2_]OH^−^ in anhydrous acetonitrile. An aliquot of the activity was incubated with 107 µg (20 nmol) of the precursor for 10 min at 70 °C, followed by cartridge purification. In general, the labeled product could be obtained within 26 min in radiochemical yields ranging from 15 to 58% d.c. and molar activities of 3–15 MBq/nmol (*n* = 4, 20 nmol precursor). Radio-HPLC and radio-TLC analysis confirmed the high radiochemical purity (> 97%) of the product.

^18^F-Labeling of **NOTA-6-Ahx-NonaLysan** occurred *via* the aluminum fluoride method in a 0.5 M NaOAc/HOAc buffer (pH 4) (Laverman et al. 2010). [^18^F]Fluoride was incubated with AlCl_3_ in ethanol/0.5 M NaOAc/HOAc buffer (1:1) (vol/vol), followed by the addition of 300 µg (55 nmol) of the chelator-conjugated peptide. The mixture was heated to 100 °C for 20 min, and the radiolabeled product was purified *via* solid phase extraction. After a total synthesis time of 43 min, Al[^18^F]F-**NOTA-6-Ahx-NonaLysan** was obtained in radiochemical yields ranging from 49 to 71% d.c. and high purity (> 97%). The molar activity was 3–5 MBq/nmol (*n* = 6, 55 nmol precursor).

### In vitro evaluation

The ^18^F-fluorinated peptides showed high stability in PBS (*n* = 1) and human blood serum (*n* = 2) (Table 2). No signs of degradation were detected by HPLC analysis for up to two hours of incubation. Protein binding was generally lower for Al[^18^F]F-**NOTA-6-Ahx-NonaLysan** compared to [^18^F]**SiFA-NonaLysan** at all time points. The *logD* values indicated higher hydrophilicity of the chelator-based peptide compared to the SiFA-derivate (-4.81 ± 0.05 vs. -2.63 ± 0.04, respectively). In addition, [^18^F]**SiFA-NonaLysan** showed increased retention on a C_18_ HPLC column compared to Al[^18^F]F-**NOTA-6-Ahx-NonaLysan** (Fig. S12). The affinity of both compounds was tested in a solid-phase-based assay. Therefore, unlabeled **SiFA-NonaLysan** and non-radioactive **AlF-NOTA-6-Ahx-NonaLysan** *(see SI)* were diluted in PBS + 0.1% BSA (10^− 5^ – 10^− 12^ M) and their potency to deplete [^125^I]iodo-asialoorosomucoid was measured. Both compounds exhibited low nanomolar affinity (IC_50_) for the immobilized ASGR1, regardless of the strategy for ^18^F-fluorination.

### In vivo evaluation

**Fig. 2:**
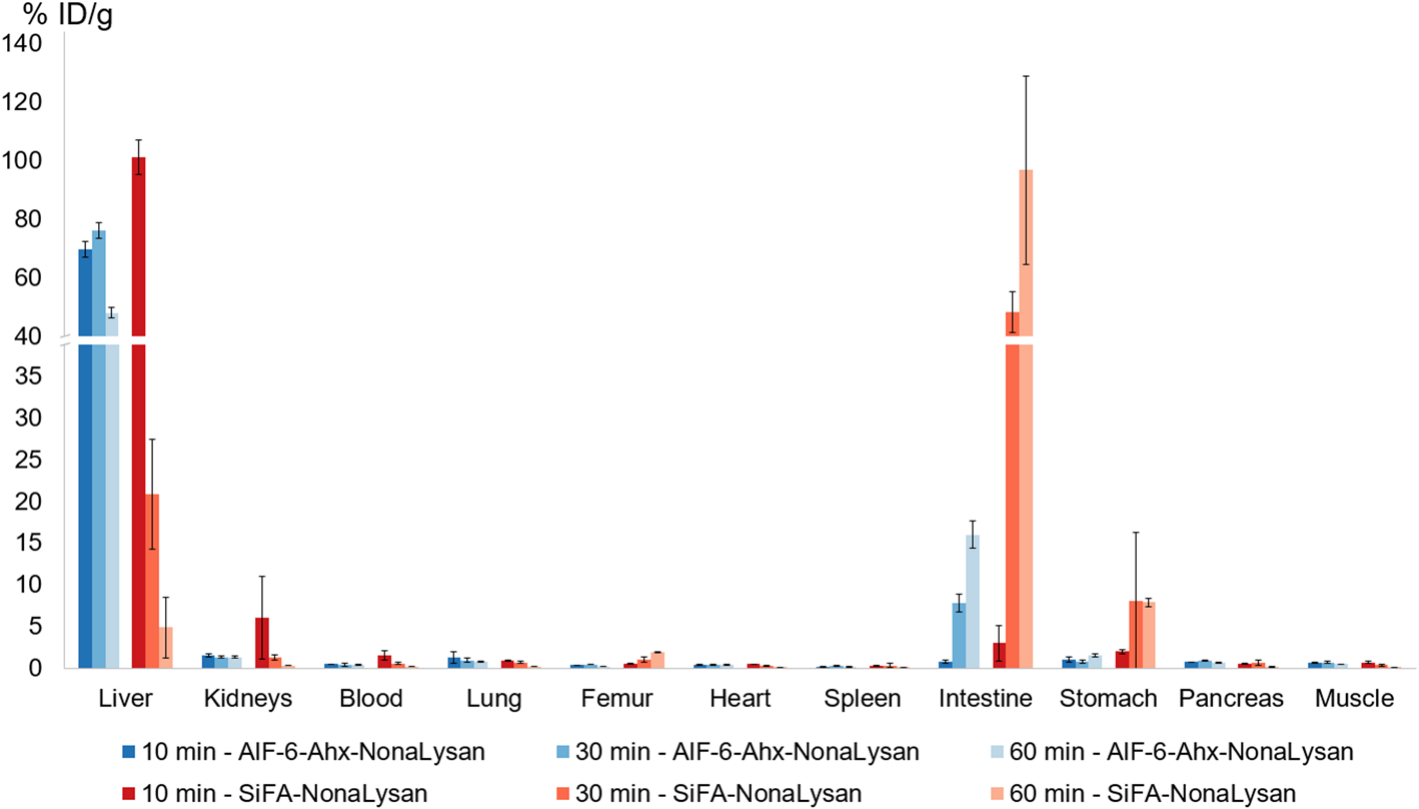
Biodistribution data (% ID/g) of Al[^18^F]F-**NOTA-6-Ahx-NonaLysan** & [^18^F]**SiFA-NonaLysan** in healthy BALB/c mice 10 (*n* = 3), 30 (*n* = 3), and 60 min (*n* = 3) p.i. (200–400 pmol, 1 MBq) depicted as mean value ± SD

**Fig. 3:**
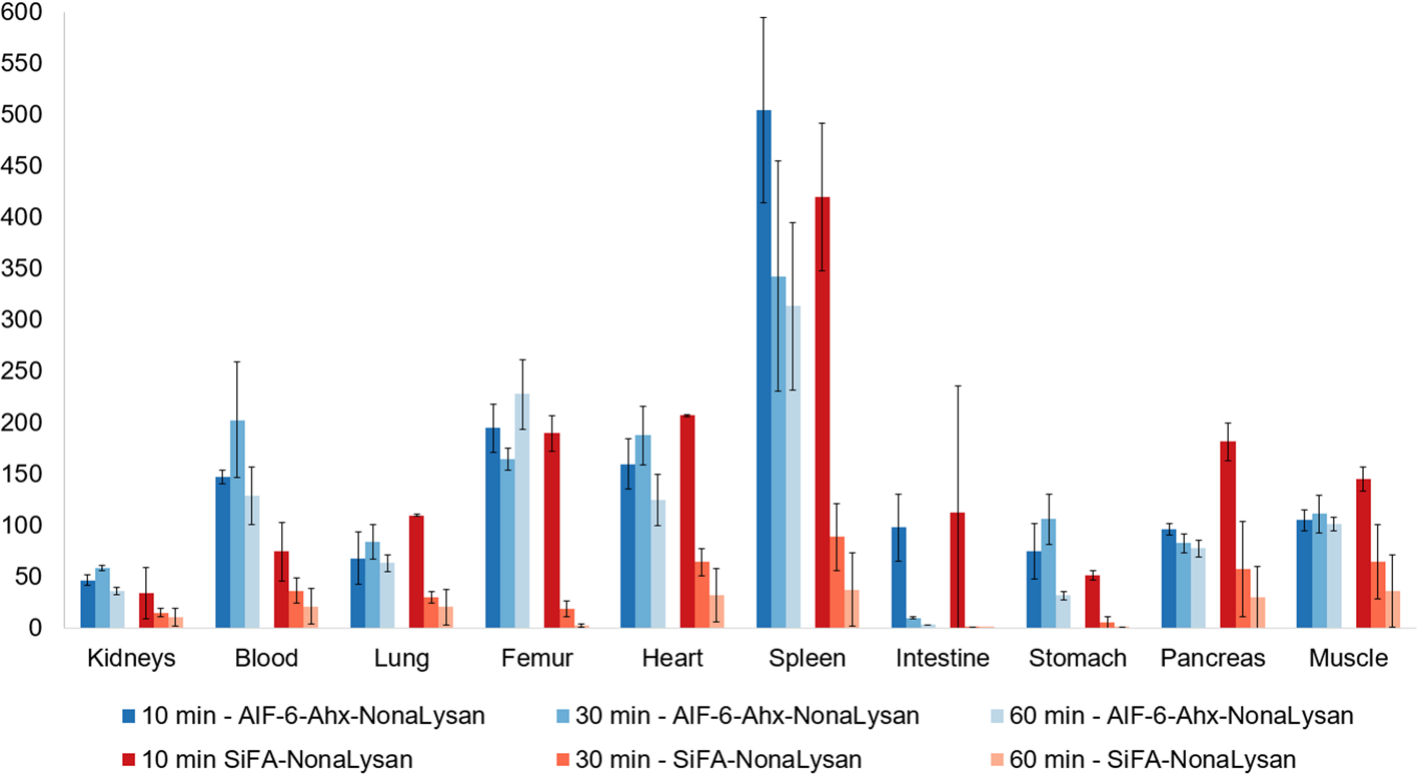
Liver-to-organ ratios of Al[^18^F]F-**NOTA-6-Ahx-NonaLysan** & [^18^F]**SiFA-NonaLysan** in healthy BALB/c mice 10 (*n* = 3), 30 (*n* = 3),and 60 min (*n* = 3) p.i. (200–400 pmol, 1 MBq) depicted as mean value ± SD

In biodistribution studies [^18^F]**SiFA-NonaLysan** exhibited the highest initial liver uptake (101.2 ± 5.8% ID/g, 10 min p.i.) (Fig. 2). However, this value dropped fast to 4.9 ± 3.6% ID/g, 60 min p.i., while at the same time a remarkable activity accumulation in the intestine was observed (96.7 ± 32.1% ID/g, 60 min p.i.). This activity shift was reflected in the liver-to-organ ratios (Fig. 3). The best contrast could be found 10 min p.i., whereas at later time points the values were significantly downshifted. In other organs, the off-target binding was minimal and the only pronounced activity accumulation was seen in the stomach starting as low as 2.0 ± 0.3% ID/g 10 min p.i. and reaching 7.9 ± 0.6% ID/g 60 min p.i.

**Table 2:** Serum and PBS stability, protein binding, *logD* & IC_50_-values of ^18^F-labeled Glycopeptides

Min	Conjugate stability in serum (% intact ligand)				Stability in PBS (% intact ligand)				Protein binding (%)				logD	IC_50_^a^ *(nM)*
	2	30	60	120	2	30	60	120	2	30	60	120		
[^18^**F]SiFA-NonaLysan**	99.0 ± 0.5	98.5 ± 0.1	99.2 ± 0.4	99.7 ± 0.2	99.1	99.1	99.2	99.2	21.8 ± 5.3	23.4 ± 2.0	19.6 ± 3.6	29.1 ± 2.0	-2.63 ± 0.04	0.9 ± 1.1
**Al[** ^18^ **F]NOTA-6-Ahx-NonaLysan**	99.9	99.9	99.9	99.9	99.9	99.9	99.9	99.9	6.3 ± 0.1	5.1 ± 0.4	7.5 ± 1.2	5.2 ± 1.7	-4.81 ± 0.05	3.0 ± 2.1

^*a*^ Half-maximum inhibitory concentration of [^125^I]iodo-asialoorosomucoid binding to the immobilized human recombinant ASGR1; for the assay the non-radioactive compounds were used.

Al[^18^F]F-**NOTA-6-Ahx-NonaLysan** reached lower liver uptake values (69.6 ± 2.8% ID/g 10 min p.i.), but the activity was longer and more stably retained in the hepatic tissue (Fig. 2). Still 47.9 ± 1.7% ID/g was found in the liver at 60 min p.i. Also, no significant activity accumulation in the stomach was detected. The overall off-target binding was minimal, and the only organ showing increasing tracer accumulation over time were the intestines (0.8 ± 0.2% ID/g 10 min p.i. to 16.0 ± 1.6%ID/g 60 min p.i.). Consequently, the liver-to-organ ratios were more constant and generally higher at later time points compared to [^18^F]**SiFA-NonaLysan** (Fig. 3). In the blocking experiment, both compounds showed a statistically significant reduction of liver uptake with only little effect on the overall off-target binding (Fig. S13). Both tracers showed reduced bone accumulation, indicating that [^18^F]fluoride is stably bound to the radiopharmaceutical.

**Fig. 4:**
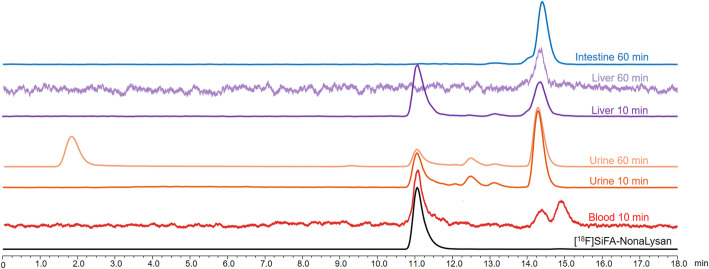
Radio-HPLC chromatograms of [^18^F]**SiFA-NonaLysan** from organ extracts; mice were injected with 5 MBq of radioligand and euthanized 10 and 60 min p.i. column: Macherey Nagel Nucleosil 120-5 C18 150 × 4.6 mm, 5 μm; solvent A: H_2_O/0.1% TFA; solvent B: MeCN/0.1% TFA; flow: 1 mL/min; Gradient: 5–60% B in 15 min

To elucidate the mechanism underlying the unexpected accumulation of intestinal activity of the SiFA-bearing radioligand, in vivo metabolite studies were performed. In case of [^18^F]**SiFA-NonaLysan**, only 57.3% of compound was still intact in the blood sample 10 min p.i. and the formation of 2 lipophilic metabolites could be observed (Fig. 4). The total amount of intact tracer was even lower in the urine sample (33.3%), where after 10 min already 4 lipophilic metabolites could be detected and after 60 min the release of free [^18^F]fluoride was visible. In the liver sample, 57% of the tracer was still intact 10 min p.i. However, at 60 min p.i., the ligand was completely metabolized, and the accumulation of a lipophilic main metabolite could be observed within the intestines.

**Fig. 5:**
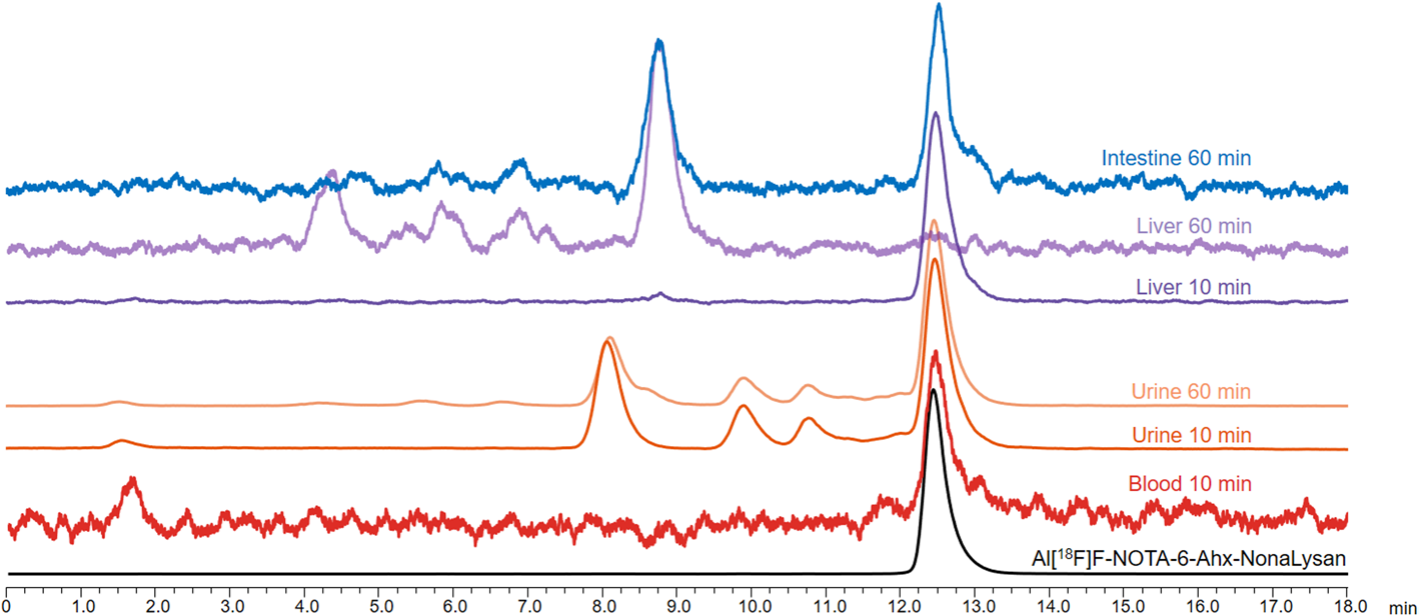
Radio-HPLC chromatograms of Al[^18^F]F-**NOTA-6-Ahx-NonaLysan** from organ extracts; mice were injected with 2.5 MBq of radioligand and euthanized 10 and 60 min p.i. column: Macherey Nagel Nucleosil 120-5 C18 150 × 4.6 mm, 5 μm; solvent A: H_2_O/0.1% TFA; solvent B: MeCN/0.1% TFA; flow: 1 mL/min; Gradient: 5–25% B in 15 min

Regarding Al[^18^F]F-**NOTA-6-Ahx-NonaLysan**, 78.8% of the tracer was still intact in the blood 10 min p.i. (Fig. 5). A higher degree of metabolization could be observed in the urine with only 47.9% of intact radioligand at 10 and 60 min p.i. There, several hydrophilic metabolites could be identified, as well as partial release of [^18^F]fluoride from the chelate. The initial liver sample at 10 min p.i. showed a high amount of intact radioligand (92.7%), but due to the formation of several hydrophilic metabolites, the value dropped to zero at 60 min p.i. The intestine sample at 60 min p.i. contained intact tracer (52.7%) as well as hydrophilic metabolites.

### Small animal imaging

 For the production of [^18^F]**SiFA-NonaLysan** for the imaging study, 456 MBq [^18^F]fluoride was incubated with 107 µg (20 nmol) of the precursor for 10 min at 70 °C. Subsequent cartridge purification afforded 201 MBq of the product, resulting in a minimum molar activity of 10 MBq/nmol. For Al[^18^F]F-**NOTA-6-Ahx-NonaLysan** 555 MBq of [^18^F]fluoride was incubated with AlCl_3_ in ethanol/0.5 M NaOAc/HOAc buffer (1:1) (vol/vol), followed by the addition of 300 µg (55 nmol) of the chelator-conjugated peptide. The mixture was heated to 100 °C for 20 min, and the radiolabeled product was purified by solid-phase extraction, yielding 81.6 MBq of the product, resulting in a minimum molar activity of 1.5 MBq/nmol.

**Fig. 6:**
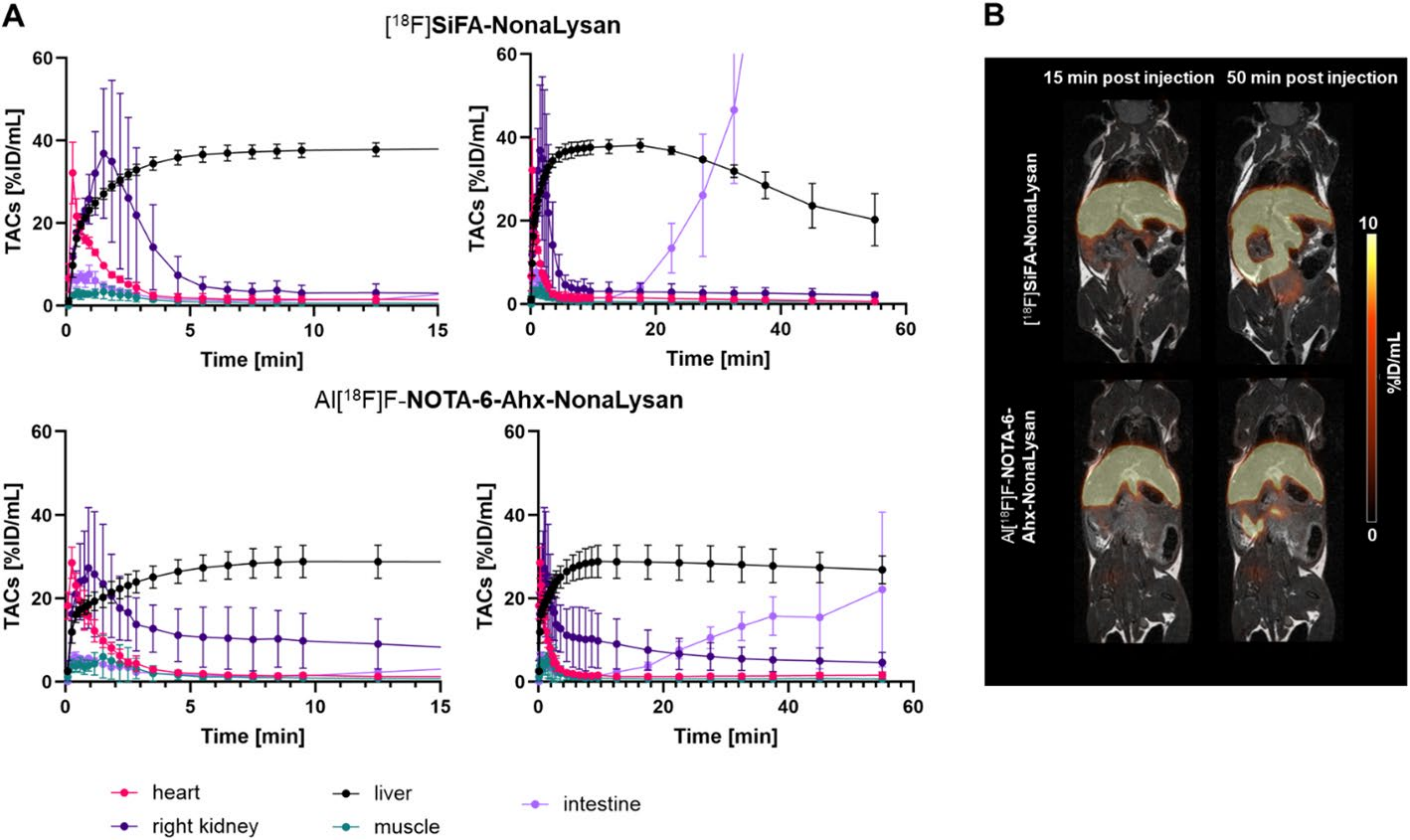
**A** Time activity curves of liver, heart, kidney, intestine, and muscle were derived from dynamic PET data for [^18^F]**SiFA-NonaLysan** (top; *n* = 3) and Al[^18^F]F-**NOTA-6-Ahx-NonaLysan** (bottom; *n* = 3). Both tracers showed stable hepatic uptake with low background activity for the first 20 min and clearance through the intestines. In case of [^18^F]**SiFA-NonaLysan**, the intestinal activity partially exceeded the liver uptake.**B** Representative images of [^18^F]**SiFA-NonaLysan** (top) and Al[^18^F]F-**NOTA-6-Ahx-NonaLysan** (bottom) for 15–20 min and 50–60 min p.i

PET/MR imaging further supported the findings of the biodistribution studies (Fig. 6). Both tracers showed a rapid increase of liver uptake in the first few minutes, reaching values of 37.6 ± 1.4%ID/mL and 28.9 ± 3.2%ID/mL after ~ 10 min for [^18^F]**SiFA-NonaLysan** and Al[^18^F]F-**NOTA-6-Ahx-NonaLysan**, respectively. [^18^F]**SiFA-NonaLysan** showed negligible background uptake but a clear washout from the liver and intestinal uptake after ~ 20 min p.i. Al[^18^F]F-**NOTA-6-Ahx-NonaLysan**, on the other hand, remained more stable over the imaging period of 60 min, with slightly increased uptake in the kidney and comparably moderate uptake in the intestines. TAC derived LHL15 was comparable at 0.96 ± 0.02 and 0.96 ± 0.01 for [^18^F]**SiFA-NonaLysan** and Al[^18^F]F-**NOTA-6-Ahx-NonaLysan**, respectively.

## Discussion

Recently, we published [^68^Ga]Ga-NODAGA-NonaLysan, a potent peptide-based galactose nonamer for PET imaging of liver function (Zierke et al. 2025). As a further development, we now synthesized and evaluated [^18^F]**SiFA-NonaLysan** and Al[^18^F]F-**NOTA-6-Ahx-NonaLysan**, two fluorinated analogues thereof. The SiFA chemistry allowed the convenient ^18^F-labeling of biomolecules under mild conditions *via* an isotopic exchange mechanism. The aluminum fluoride formation required more elevated temperatures and longer reaction times, but worked well with a chelator-conjugated targeting vector. In general, both radiolabeling strategies were suitable for the ^18^F-fluorination of peptides. However, when compared to classical ^68^Ga-labeling procedures, substantial differences regarding the amount of precursor, the pH, the temperature, the overall reaction time, and the radiochemical yields became apparent. Manual ^68^Ga-labeling of NODAGA-NonaLysan usually requires only 5 nmol of peptide and proceeds within 15 min at 95 °C and pH 4 in quantitative yield (Zierke et al. 2025). In contrast, the ^18^F-fluorination *via* the SiFA strategy required 20 nmol of precursor. The reaction proceeded at neutral pH and in anhydrous acetonitrile for 10 min at 70 °C. The highest possible radiochemical yield was 58%, consistent with literature data (Wängler et al. 2010; Deiser et al. 2024). Labeling *via* aluminum fluoride formation required even higher amounts (55 nmol) of precursor. The complexation of Al[^18^F]F occurred within 20 min at 100 °C and pH 4 with a maximum yield of 71%. This value lay in the medium range according to literature (D’Souza et al. 2011; Laverman et al. 2012). The necessity of such large precursor amounts has also been observed for other AlF-labelled radioconjugates and is reported as necessary to obtain sufficient radiochemical conversion (Kersemans et al. 2018; Yu et al. 2020). Other parameters that deserved attention were the pH, the aluminum-to-precursor ratio, and the solvent composition. Like the metal complexation of gallium-68, there is also a strong pH dependency, and the optimum pH lies between 4.0 and 4.5 (Kang et al. 2021). Preconditioning the QMA cartridge for ^18^F-fixation with the reaction buffer was essential to obtaining the correct pH range for the reaction. Regarding the aluminum concentration, a molar ratio of 1:2 (Al^3+^/precursor) has been reported to be optimal. Furthermore, the addition of an organic cosolvent such as ethanol can significantly increase the labeling yield (Kersemans et al. 2018; Kang et al. 2021). Also, the choice of the chelator can have an influence on the reaction outcome. NOTA is reported to provide higher yields than NODAGA due to the absence of an additional carboxylate, which is believed to compete with the coordination site of fluoride on the aluminum surface (Fersing et al. 2019, Kang et al. 2021).

Regarding the in vitro evaluation, both fluorinated peptides showed high stability in human blood serum and PBS for up to 2 h of incubation, which is comparable to the values found for [^68^Ga]Ga-NODAGA-NonaLysan (Zierke et al. 2025). The amount of protein binding was low for Al[^18^F]F-**NOTA-6-Ahx-NonaLysan**, but significantly elevated for [^18^F]**SiFA-NonaLysan.** This phenomenon was somehow expected as replacing the chelator with the lipophilic SiFA can enhance non-specific interactions (Wängler et al. 2012). The *logD* values reflected this finding, and the SiFA-bearing ligand showed a 100-fold higher accumulation in the octanol phase. In addition, when both compounds were analyzed by radio-HPLC with the same gradient, [^18^F]**SiFA-NonaLysan** exhibited stronger interaction with the C_18_ column material. Despite these differences, both compounds maintained a low nanomolar affinity for the ASGR. One could conclude that the choice of the ^18^F-labeling strategy did not inherently alter the in vivo uptake properties. However, the biodistribution data spoke a different language.

[^18^F]**SiFA-NonaLysan** reached the highest liver uptake of all compounds we have investigated so far (Zierke et al. 2024a, b; Zierke et al. 2024a, b, 2025), including Al[^18^F]F-**NOTA-6-Ahx-NonaLysan**. In contrast to the chelator-based derivatives, activity was poorly retained in hepatic tissue and accumulated rapidly in the intestine and the stomach. Consequently, the liver-to-organ ratios dropped strongly, and thus, 30 min p.i., only 1/5 of the maximum accumulated activity was still found in the liver. Overall, the different pharmacodynamic behavior of both tracers was confirmed by the PET/MR studies. Again, the elimination from the liver into the intestines was much more pronounced for [^18^F]**SiFA-NonaLysan**. However, the elimination rate from the liver was a lot lower than found in the biodistribution studies. One explanation might be that during PET/MR imaging, the mice remained anesthetized over the whole imaging period of 60 min. In the biodistribution study, mice were awake between tracer injection and the time point of investigation. Anesthetics such as Isoflurane have been reported to alter the metabolization rates of the tested compounds (Hildebrandt et al. 2008).

Nevertheless, this result was unexpected, as we had estimated that the presence of 9 galactose moieties would effectively counterbalance the lipophilic SiFA group. Indeed, unwanted hepatic and gastrointestinal uptake is a common artefact of SiFA-bearing radioligands and often requires the conjugation of hydrophilic linkers or chelators (Wängler et al. 2010; Wurzer et al. 2020; Bailey et al. 2021). To ensure that the liver uptake of [^18^F]**SiFA-NonaLysan** and Al[^18^F]F-**NOTA-6-Ahx-NonaLysan** was receptor-specific, a blocking experiment was conducted at the earliest time point. Co-injection of an excess of GSA led in both cases to a statistically significant reduction of the hepatic uptake. Therefore, we assumed that the origin of the different biodistribution pattern must occur further downstream in the internalization cascade.

Both compounds presumably bind to the receptor as agonists and undergo immediate internalization. Consequently, they are transferred into the lysosome, where the receptor is separated from the ligand and gets recycled (Connolly et al. 1982). The ligands get either degraded or excreted *via* the bile ducts into the intestines, as also seen for [^99m^Tc]Tc-GSA (Zierke et al. 2024a, b). In case of [^18^F]**SiFA-NonaLysan**, a lipophilic radioactive metabolite is formed, which follows excretion preferably into the bile and hence ends up in the intestines. In contrast, the biodistribution of Al[^18^F]F-**NOTA-6-Ahx-NonaLysan** is similar to that of [^68^Ga]Ga-NODAGA-NonaLysan. Upon internalization, the radioligand gets metabolized *via* hydrophilic, possibly charged chelator-conjugated species, which are better retained within the cell.

The question now is whether these pharmacodynamic differences have a significant influence on the determination of the functional liver mass. It is known that other radiotracers such as [^99m^Tc]Tc-Mebrofenin (de Graaf et al. 2010) or [^68^Ga]Ga-TEoS-DAZA (Greiser et al. 2018) also follow immediate elimination into the bile ducts and are of great value for hepatobiliary scintigraphy. More importantly, some of the clinical parameters used in the [^99m^Tc]Tc-GSA scintigraphy to determine the functional liver mass, as the LHL15 value, are calculated already in the early phase of the tracer uptake in the liver. As demonstrated, the LHL15 values (0.96 ± 0.02 vs. 0.96 ± 0.01) are very high and comparable for both investigated compounds, indicating comparable diagnostic quality. However, as we believe that a stable activity retention in the functional hepatic tissue eases the interpretability of the PET/MR images, Al[^18^F]F-**NOTA-6-Ahx-NonaLysan** might be the fluorinated tracer of choice. At the end, further studies in animal disease models or even first-in-human studies would be necessary to confirm the superiority of one of the compounds.

## Conclusion

In this study, we successfully synthesized two new ^18^F-labeled radiopharmaceuticals targeting the ASGR. Despite their similar chemical structure, remarkable differences in their pharmacokinetic profile became apparent. [^18^F]**SiFA-NonaLysan** reached an extremely high liver uptake in the initial phase, but was rapidly excreted from the target organ. In contrast, Al[^18^F]F-**NOTA-6-Ahx-NonaLysan** showed a lower but steadier liver uptake pattern, similar to the lead compound [^68^Ga]Ga-NODAGA-NonaLysan, indicating the superior imaging properties of Al[^18^F]F-**NOTA-6-Ahx-NonaLysan**.

## Supplementary Information

Below is the link to the electronic supplementary material.

Supplementary Material 1

## Data Availability

The datasets generated during the present study are available from the corresponding author upon reasonable request.

## Abbreviations

AlF: Aluminium fluoride species
A_m_: Molar activity
ASGR: Asialoglycoprotein receptor
ASOR: Asialoorosomucoid
d.c.: Decay corrected
DIPEA: Diisopropylethylamine
DMF: Dimethylformamide
DMSO: Dimethylsulfoxide
GSA: Galactosyl serum albumin
Fig.: Figure
HATU: [O-(7-Azabenzotriazol-1-y)-N, NN’N’-tetramethyluronium-hexafluorophosphat
HOAc: Acetic acid
HOAt: 1-hydroxy-7-azabenzotriazol
HPLC: High performance liquid chromatography
IC_50_: Inhibitory constant
ID/g: Injected dose per gram
KOH: Potassium hydroxide
MeCN: Acetonitrile
MeOH: Methanol
MRI: Magnetic resonance imaging
NaOAc: Sodium acetate
NEt_3_: Triethylamine
NHS: N-hydroxy succinimide
NOTA: 1,4,7-triazacyclononane-1,4,7-triacetic acid
o.n.: Over night
p.i.: Post injection
PBS: Phosphate-buffered saline
PET: Positron emission tomography
SD: Standard deviation
SI: Supplementary Information
SiFA: Silicon fluoride acceptor
TAC: Time activity curve
tBuOH: TertButanol
TFA: Trifluoroacetic acid
TIPS: Triisopropylsilan
TLC: Thin-layer chromatography
t_R_: Retention time
WHO: World Health Organization
