# Supplementary material for: Comparison of two 18F-fluorinated glycopeptides for PET imaging of the functional liver mass
Source: EJNMMI Radiopharm Chem. 2026 Apr 4;11:35. doi: 10.1186/s41181-026-00445-z (PMC13187097; doi:10.1186/s41181-026-00445-z)
Supplement: Supplementary file 1 — Supplementary Material 1 [file 41181_2026_445_MOESM1_ESM.docx]

**Supporting Information**

Comparison of two ^18^F-Fluorinated Glycopeptides for PET Imaging of the Functional Liver Mass

Maximilian A. Zierke^1^, Katharina Hofer^1^, Kimia Samadikhah^2^, Christine Rangger^1^, Carmen Wängler^3,4^, Björn Wängler^4,5^, Anna Junker^2,6^, Andreas M. Schmid^2,6^, Roland Haubner^1,§^

^1^Department of Nuclear Medicine, Medical University Innsbruck, Anichstr. 35, 6020 Innsbruck, Austria

^2^Werner Siemens Imaging Center, Department of Preclinical Imaging and Radiopharmacy, Eberhard Karls University Tübingen, Röntgenweg 13, 73076 Tübingen, Germany

^3^Biomedical Chemistry, Clinic of Radiology and Nuclear Medicine, Medical Faculty Mannheim, Heidelberg University, Theodor-Kutzer-Ufer 1-3, 68167 Mannheim, Germany

^4^Research Campus M²OLIE, Medical Faculty Mannheim, Heidelberg University, 68167 Mannheim, Germany;

^5^Molecular Imaging and Radiochemistry, Clinic of Radiology and Nuclear Medicine, Medical Faculty Mannheim, Heidelberg University, Theodor-Kutzer-Ufer 1-3, 68167 Mannheim, Germany

^6^Cluster of Excellence iFIT (EXC 2180) “Image-Guided and Functionally Instructed Tumor Therapies”, University of Tübingen, 73076 Tübingen, Germany

§to whom correspondence should be addressed

Roland Haubner, PhD, Medical University Innsbruck. E-mail: [roland.haubner@i-med.ac.at](mailto:roland.haubner@i-med.ac.at)

**Table of Content**

[**1.** **General Procedures for Solid Phase Peptide Chemistry** 2](#_Toc224292729)

[**2.** **Synthetic Procedures and Analytical Data** 4](#_Toc224292730)

[*2.1 Fmoc-K(6-heptynoic acid)-GG-OH* 5](#_Toc224292731)

[*2.2 SiFA-NonaLysan* 6](#_Toc224292732)

[*2.3 H-6-Ahx-NonaLysan* 9](#_Toc224292733)

[*2.4 NOTA-6-Ahx-NonaLysan* 11](#_Toc224292734)

[*2.5 Nonradioactive AlF-NOTA-6-Ahx-NonaLysan* 12](#_Toc224292735)

[**3.** **Description of the material used for the preparation of the tracer used for the PET imaging studies** 14](#_Toc224292736)

[**4.** **Additional Tables and Figures** 15](#_Toc224292737)

# **General Procedures for Solid Phase Peptide Chemistry**

*Loading of fragments on to a Rink amide resin (GP1)*

Rink amide resin (80 mg, δ = 0.65 mmol/g, 1.0 eq) was weighed into a 10 mL syringe equipped with a pp-frit inlet and was swollen in DMF for 1 hour. Removal of the first Fmoc-protection group was accomplished by incubating the resin for 1 hour with a mixture of 20 % Pip/DMF (vol/vol). The resin was washed with DMF (8 × 3 mL) and a solution of 0.9 eq Fmoc-K(6-heptynoic acid)GG-OH, 0.9 eq HOAt, 0.9 eq HATU and 4.5 eq DIPEA in DMF was added. After 24 hours of incubation time the resin was washed with DMF (3 × 3 mL) and all non-reacted active sites were capped by adding a mixture of Acetic Anhydride/Pyridine (3:2) (vol/vol) for 30 min. The resin was washed once again with DMF (3 × 3 mL) and was ready to use for the next step.

*On-resin peptide bond formation for single amino acids (GP2a)*

In a 5 mL glass vial HOAt (2.0 eq), HATU (2.0 eq) and the respective amino acid (2.0 eq) were dissolved in 3 mL of DMF with stirring. DIPEA (6.0 eq) was added and the solution was stirred for another 5 min at room temperature. The mixture was added to the resin and the reaction was allowed to continue for 2 hours.

*Fragment Coupling* *(GP2b)*

Fmoc-K(6-heptynoic acid)GG-OH (1.0 eq) was weighed into a 5 mL glass vial together with 1.0 eq HOAt and 1.0 eq HATU. DMF (2 mL) and DIPEA (6.0 eq) were added and the solution was stirred for 5 min at room temperature. This mixture was then added to the resin and the reaction was allowed to continue for 3 hours.

*Fmoc-Removal (GP3)*

The resin was treated with a mixture of 20 % Pip/DMF (vol/vol) (1 × 5 min, 1 × 15 min) and was washed with DMF afterwards. (8 × 6 mL/g resin).

*On-resin Dde-deprotection* (*GP4*)

For on resin Dde-deprotection in the presence of Fmoc-groups the resin was treated with a solution of Imidazole (0.92 g/g resin) and Hydroxylamin Hydrochloride (1.26 g/g resin) in 5 mL of NMP and 1 mL of DMF. The solution was added to the resin for a time period of 3 hours and the resin was washed with DMF (3 × 6 mL/g resin) afterwards.

*Capping of unreacted amines (GP5)*

The resin was treated for 30 min with 5 mL of a freshly prepared Acetic Anhydride/Pyridine (3:2) solution. Afterwards the resin was washed 3 times with DMF.

*Cleavage of peptides from the resin* *(GP6)*

The resin was washed thoroughly with DCM (3 × 6 mL) before 3 mL of a cleavage cocktail containing TFA/TIPS/H_2_O (95/2.5/2.5) (vol/vol/vol) were added. After 45 min the solution containing the crude peptide was transferred into a 15 mL round bottom flask and 2 mL of new cleavage cocktail were added to the syringe. This step was repeated three times in total. The combined fractions were reduced under a stream of Argon and either precipitated in ice-cold diethylether or directly lyophylized in a mixture of H_2_O*/tBu*OH (1:1) (vol/vol).

# **Synthetic Procedures and Analytical Data**

**S1.** Preparation of **NOTA-6-Ahx-NonaLysan** & **SiFA-NonaLysan** by a fragment condensation approach. Fmoc-K(6-heptynoic acid)GG-OH is immobilized on a Rink amide resin and serves as the general building block for chain elongation. Glycosylation and attachment of NOTA is performed in solution after cleavage from the solid support. SiFA-benzoic acid is coupled on resin, followed by cleavage of the peptide and glycosylation in solution.

a – Pip/DMF (20 %); b – Fmoc-K(6-heptynoic acid)GG-OH, HOAt, HATU, DIPEA (DMF); c – Pip/DMF (20 %); d – Fmoc-GABA-OH, HATU, DIPEA (DMF); e – Pip/DMF (20 %); f – SiFA-benzoic acid, HOAt, HATU, DIPEA (DMF); g – TFA/TIPS/H_2_O; h – 1-azido-1-deoxy-β-d-galactopyranoside, Cu(OAc)_2_, Na-ascorbate (*t*BuOH/H_2_O); i – Pip/DMF (20 %); j – Fmoc-6-Ahx-OH, HATU, DIPEA (DMF); k – Pip/DMF (20 %); l – TFA/TIPS/H_2_O; m – 1-azido-1-deoxy-β-d-galactopyranoside, Cu(OAc)_2_, Na-ascorbate (*t*BuOH/H_2_O); n – NOTA-NHS, DIPEA (DMSO).

## *2.1 Fmoc-K(6-heptynoic acid)-GG-OH*

Assembly of the alkyne tagged KGG-motif, which serves as the general building block, was achieved via solid phase peptide synthesis starting with 500 mg (550 µmol) of glycine-loaded *CTC*-resin (δ = 1.10 mmol/g). Coupling of glycine and Dde-protected l-lysine was performed accordingly to a general procedure *(GP2a & GP3)*. On-resin Dde-removal was carried out using Imidazole and Hydroxylammoniumchloride *(GP4)*. Attachment of 6-heptynoic acid to the lysine sidechain was accomplished using HOAt (1.2 eq), HATU (1.2 eq) and DIPEA (3.0 eq) over the course of 3.5 hours. Cleavage of the motif from the resin was done according to *GP6*. Purification via semi-preparative HPLC (43-55 % B in 25 min) and subsequent lyophylization yielded 316 mg (536 µmol, 87 %) of a colorless solid.

**RP-HPLC** (20-80 % B in 15 min) t_R_ = 9.0 min (52 % B).

**MALDI-MS** (m/z) = 591.5 [M+H]^+^, 613.5 [M+Na]^+^, 629.5 [M+K]^+^, 651.4 [M+Na+K]^+^.


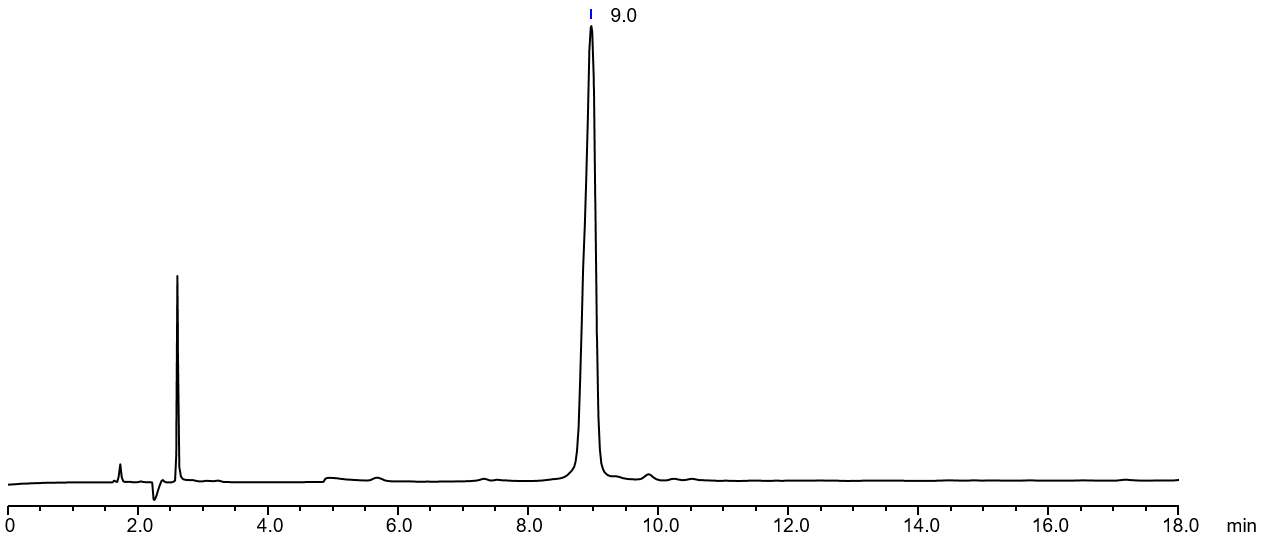


**S2.** RP-HPLC of *Fmoc-K(6-heptynoic acid)-GG-OH* at λ = 220 nm.

**S3.** Mass spectrum of *Fmoc-K(6-heptynoic acid)-GG-OH*.

## *2.2 SiFA-NonaLysan*

Fmoc-Gly Rink amide resin (110 mg, loading: 0.593 mmol/g) was swelled in DMF for one hour in a 10 mL disposable syringe with an integrated polypropylene filter. The initial Fmoc-deprotection was accomplished using a mixture of 20 % Pip/DMF for 1 hour. Subsequent coupling of Fmoc-Gly-OH and Fmoc-l-Lys(Dde)-OH followed *GP2a & GP3.* On-resin Dde-removal and the attachment of 6-heptynoic acid to the lysine-sidechain were performed according to *GP4 & GP2a*. After the removal of the Fmoc-protection group (*GP3*), the peptide chain was elongated *via* fragment coupling of Fmoc-K(6-heptynoic acid)GG-OH (*GP2b*). In between each fragment coupling, a capping step was included to avoid the formation of truncated peptide species (*GP5*). Fmoc-GABA-OH was introduced as a spacer at the *N*-terminus (*GP2a*) with an extended reaction time of three hours. The conjugation of 4-(di-*tert*-butylfluorosilyl)benzoic acid required 1.2 equivalents of HOAt, HATU, and 6.0 equivalents of DIPEA (*GP2b & GP3*). Cleavage of the peptide from the resin followed *GP6*. The residue was dissolved in 3 mL of *t*BuOH/H_2_O (1:1) (vol/vol) and was lyophilized. For glycosylation, the crude peptide (130 mg, 1,0 eq) was dissolved in 1.2 mL *t*BuOH/H_2_O (1:1) (vol/vol) together with 100 μL DMSO. To this, solutions of 1-azido-1-deoxy-β-d-galactopyranoside (90 mg, 11.8 eq) in 300 μl H_2_O, copper acetate pentahydrate (15 mg, 1.2 eq) in 300 μL H_2_O and sodium ascorbate (204 mg, 16.0 eq) in 400 μL H_2_O were added. The mixture was incubated in a water bath at 40 °C for 45 min. After centrifugation (6000 rpm for 5 min), the supernatant was purified *via* semi-preparative HPLC (27-34 % B in 25 min). Lyophylization yielded the product as a colorless solid (8.1 mg, 1.5 µmol, 2.3 %).

**Analytical HPLC** (ReproSil Pur, 5-60 % B in 15 min, 1.0 mL/min) t_R_ = 12.4 min (47 % B).

**ESI-MS**(m/z) = 1788.27 [M+3H]^3+^, 1341.70 [M+4H]^4+^, 1073.92 [M+5H]^5+^, 895.16 [M+6H]^6+^, 359.24 [M+15H]^15+^.


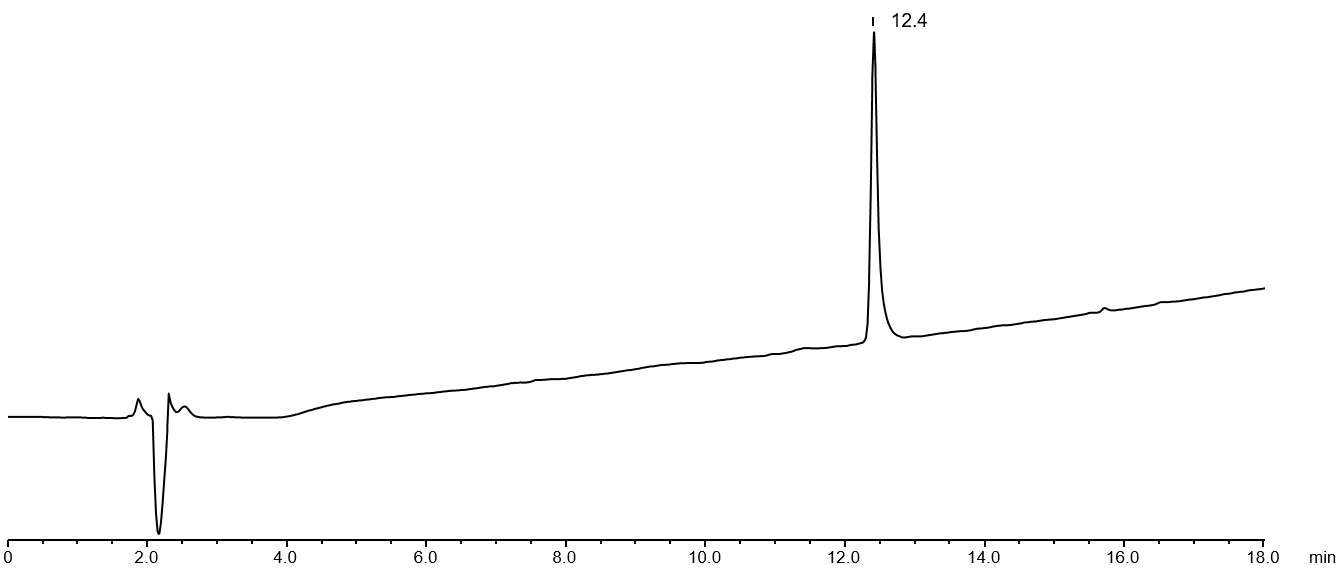


**S4.** RP-HPLC of *SiFA-NonaLysan* at λ = 220 nm.


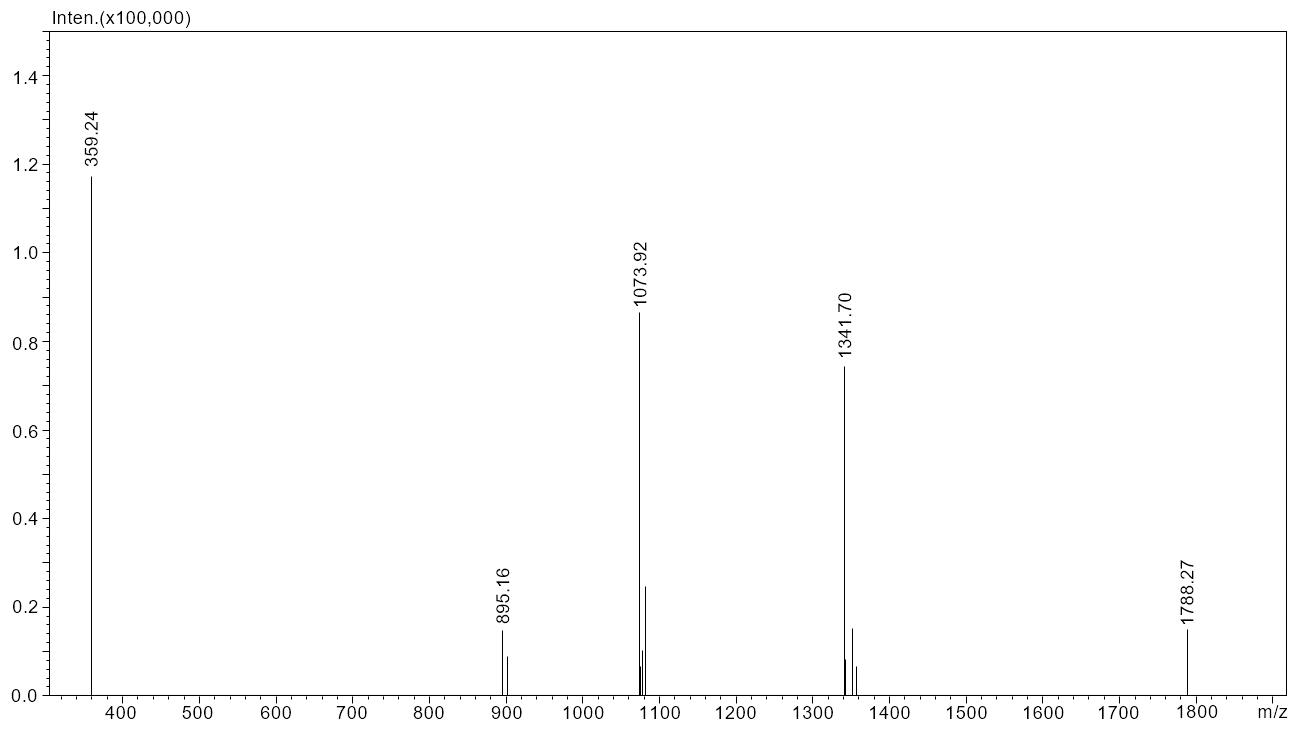


**S5.** ESI-MS (positive mode) of *SiFA-NonaLysan*.

## *2.3 H-6-Ahx-NonaLysan*

Fmoc-K(6-heptynoic acid)-GG-OH was loaded onto a Rink amide resin *(GP1),* followed by a Fmoc-deprotection step *(GP2).* Assembly of all building blocks was performed according to *GP2b* and *GP3*. After each coupling, a capping step was performed *(GP5)* to avoid the formation of truncated peptide species. After coupling of Fmoc-6-Aminohexanoic acid (*GP2b & GP3*), the Fmoc-deprotected peptide was cleaved from the resin and directly lypohilized (*GP6*).

For glycosylation, the crude peptide (20 mg, 6 µmol, 1.0 eq) was reconstituted in 300 µL *t*BuOH and mixed with an aqueous solution of 1-Azido-1-desoxy-β-d-galactopyranoside (15.5 mg, 62 µmol, 10.4 eq). Next, a catalytic amount of Cu(OAc)_2_ (360 µg, 1.8 µmol, 0.3 eq) in 36 µL Millipore water was added. The reaction started upon the addition of 13 mg (66 µmol, 11 eq) sodium ascorbate in 200 µL millipore water. After 30 min at 60 °C the mixture was centrifuged (5 min, 15 000 rpm). The supernatant was diluted with 500 µL of millipore water and purified *via* semipreparative HPLC (10-17 % B in 25 min). After lyophilisation, the product was obtained as a colorless solid (12.5 mg, 2.4 µmol, 40 %).

**Analytical HPLC** (ReproSil Pur, 5-25 % B in 15 min, 1.0 mL/min) t_R_ = 12.2 min (20 % B)

**ESI-MS** (m/z) = 1724.35 [M+2Na+1H]^3+^, 1283.55 [M+4H]^4+^, 1027.11 [M+5H]^5+^, 856.43 [M+6H]^6+^.


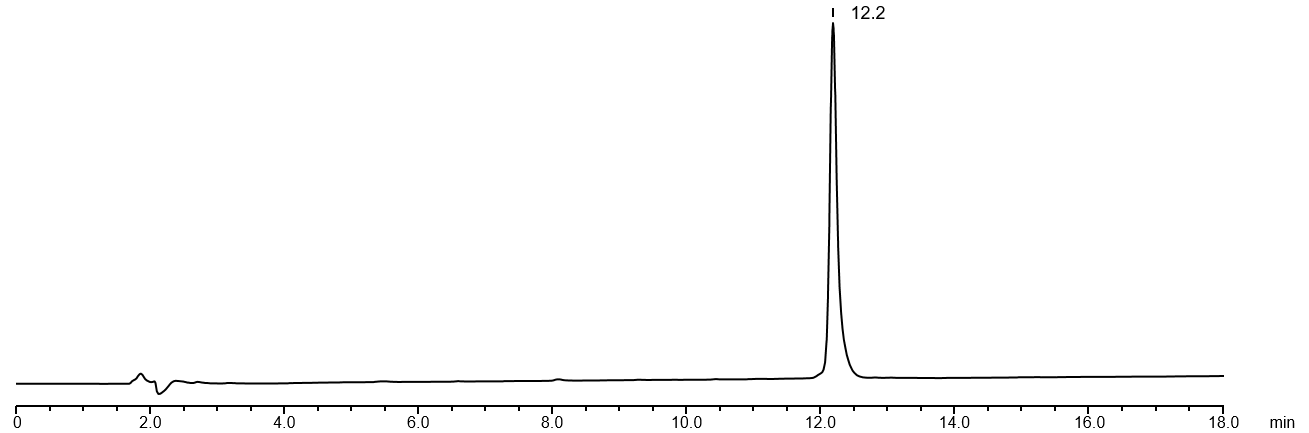


**S6.** RP-HPLC of *H-6-Ahx-NonaLysan* at λ = 220 nm.

**S7.** ESI-MS (positive mode) of *H-6-Ahx-NonaLysan*.

## *2.4 NOTA-6-Ahx-NonaLysan*

H-6-Ahx-NonaLysan (12.5 mg, 2.4 µmol, 1.0 eq) was dissolved in 300 µL anhydrous DMSO and 4.5 µL DIPEA. To this mixture, NOTA-NHS ester (4.7 mg, 7.2 µmol, 3.0 eq) in 100 µL dry DMSO was added. The pH was adjusted to 8 by another 3.5 µL DIPEA and the reaction was allowed to progress for 3 hours at room temperature with stirring. The reaction was stopped upon the addition of 500 µL millipore water and all volatiles were removed *in vacuo*. The residue was dissolved in 800 µL millipore water and subjected to purification *via* semipreparative HPLC (12-17 % B in 25 min). After lyophilization the product was obtained as a colorless solid (9.5 mg, 1.8 µmol, 73 %).

**Analytical HPLC** (ReproSil Pur, 5-60 % B in 15 min, 1.0 mL/min) t_R_ = 7.7 min (30 % B)

**ESI-MS** (m/z) = 1806.27 [M+3H]^3+^, 1354.59 [M+4H]^4+^, 1084.04 [M+5H]^5+^, 903.55 [M+6H]^6+^, 774.50 [M+7H]^7+^.


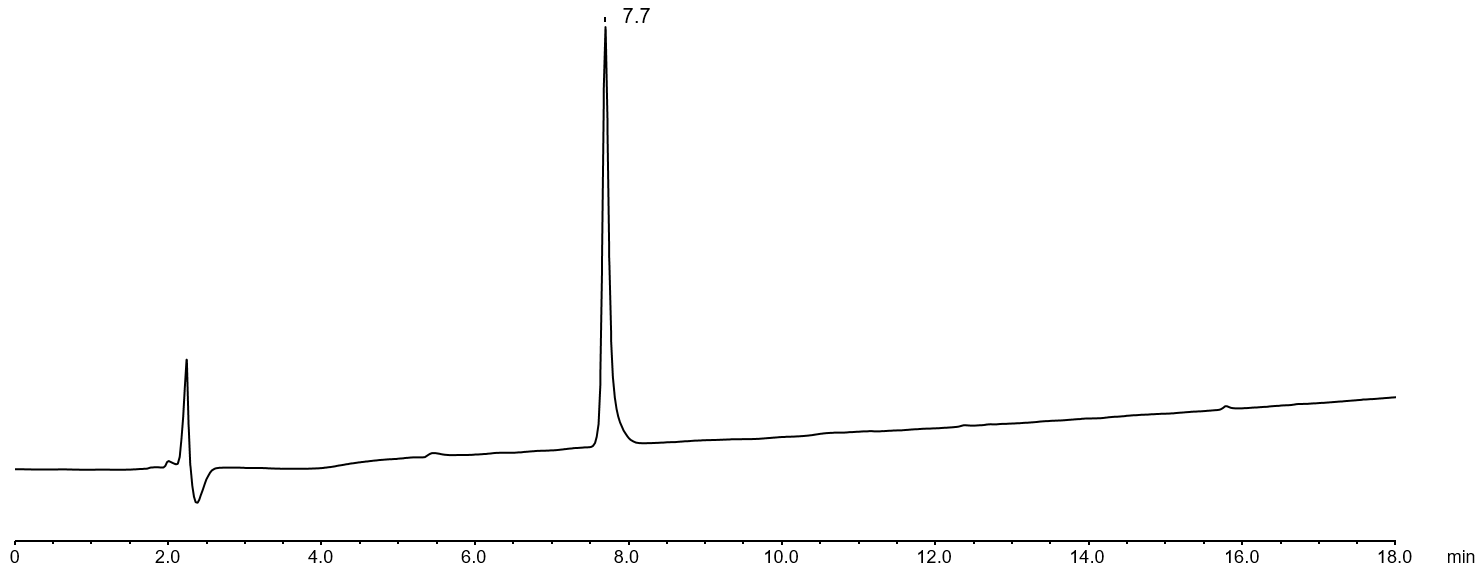


**S8.** RP-HPLC of *NOTA-6-Ahx-NonaLysan*.

**S9.** ESI-MS (positive mode) of *NOTA-6-Ahx-NonaLysan*.

## *2.5 Nonradioactive AlF-NOTA-6-Ahx-NonaLysan*

Solutions of AlCl_3_ (2.4 µmol, 20 mM, 120 µL) and KF (2.4 µmol, 20 mM, 120 µL) in 0.5 M NaOAc/HOAc-buffer pH 4 were mixed with 240 µL pure ethanol. This cocktail was incubated for 5 min at room temperature, before 1.3 mg (240 nmol) of NOTA-6-Ahx-NonyLysan was added. After heating to 100°C for 20 min, precipitates were spun off (14500 rcf, 5 min) and the supernatant was diluted 1:5 with millipore water. The nonradioactive AlF-complex was isolated *via* HPLC (14 % B isocratic). After lyophylization the product was obtained as a colorless solid (800 µg, 146 nmol, 61 %).

**Analytical HPLC** (ReproSil Pur, 5-25 % B in 15 min, 1.0 mL/min) t_R_ = 12.6 min (20 % B)

**ESI-MS** (m/z) = 1365.42 [M+4H]^4+^, 1092.90 [M+5H]^5+^, 614.33 [M+3Na+6H]^9+^.


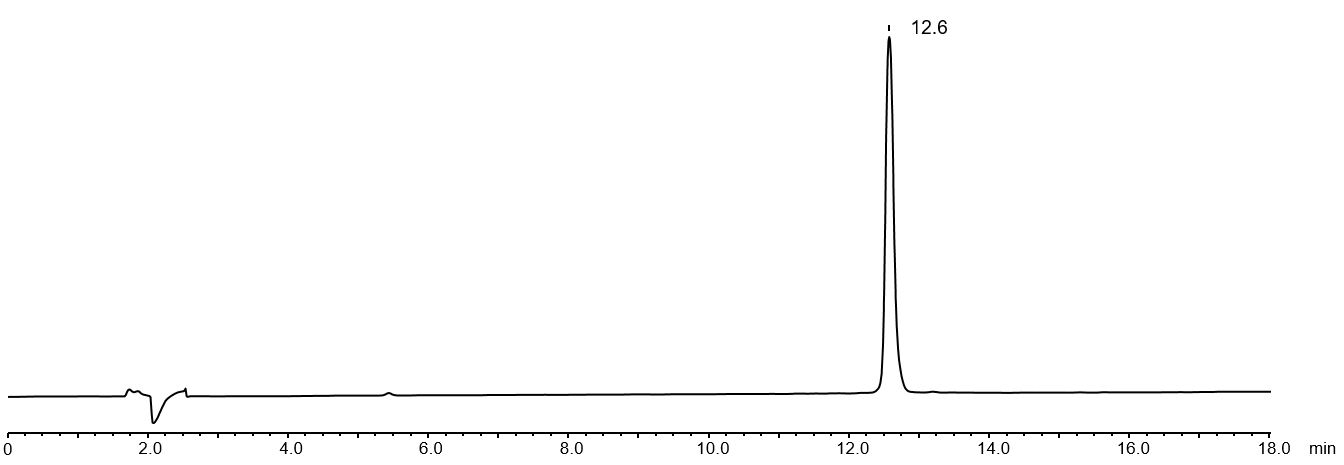


**S10.** RP-HPLC of nonradioactive *AlF-NOTA-6-Ahx-NonaLysan*.

**S11.** ESI-MS (positive mode) of nonradioactive *AlF-NOTA-6-Ahx-NonaLysan.*

# **Description of the material used for the preparation of the tracer used for the PET imaging studies**

Fluorine-18 was produced via the bombardment of [^18^O]H_2_O via the ^18^O(p,n)^18^F nuclear reaction using a PETtrace 890 (16 MeV protons) cyclotron (GE Healthcare, Uppsala, Sweden) and was delivered either as a target wash in H_2_O (1.5-2.5 mL, 0.4-1.8 GBq/mL) for manual radiochemical experiments. Manual radiochemical experiments were performed using sealable single-use borosilicate glass reaction tubes (PYREX® 9 ml, corning, New York, NY, USA) with screw-top PTFE-lined caps. All reactions were stirred using either Teflon® or glass coated micro stirrer bars. Radiochemical reaction performance was monitored using radioTLC on 0.20 mm Polygram SIL G/UV_254_ (silica gel 60) TLC plates. RadioTLC plates were developed with an appropriate running buffer/solvent mixture. RadioTLC data was acquired using a Cyclone Plus storage phosphor imaging system (PerkinElmer, Waltham, Massachusetts, USA). Analytical radio-HPLC data was collected using an Agilent HPLC (1260 Infinity series with an automated sample injector) coupled to an inline radiation detector (NaI(Tl)). In all cases, analytical radioHPLC data was obtained under the following general conditions unless otherwise stated: Column: Luna 5 μm C18 (2) 100 Å column (250 x 4.6 mm), flow 1mL/min. The following gradient was run for **SiFA-NonaLysan**: Solvent A: H_2_O + 0.1% TFA; Solvent B: MeCN; 0 - 2 min: (5% B); 0-17 min: (5 - 100% B); 17 - 23 min: (100% B); 23-28 min: (100-5% B), 28-30 min (5% B). The following gradient was run for Al[^18^F]F-**NOTA-6-Ahx-NonaLysan**: Solvent A: H_2_O + 0.1% TFA; Solvent B: MeCN; 0 - 2 min: (5% B); 0-17 min: (5 - 25% B); 17 - 23 min: (100% B); 23-28 min: (100-5% B), 28-30 min (5% B). For all radiochemical experiments, reagents, solvents, QMA eluents, reaction mixtures, and buffers were freshly prepared and dispensed directly before use unless otherwise stated.

# **Additional Tables and Figures**


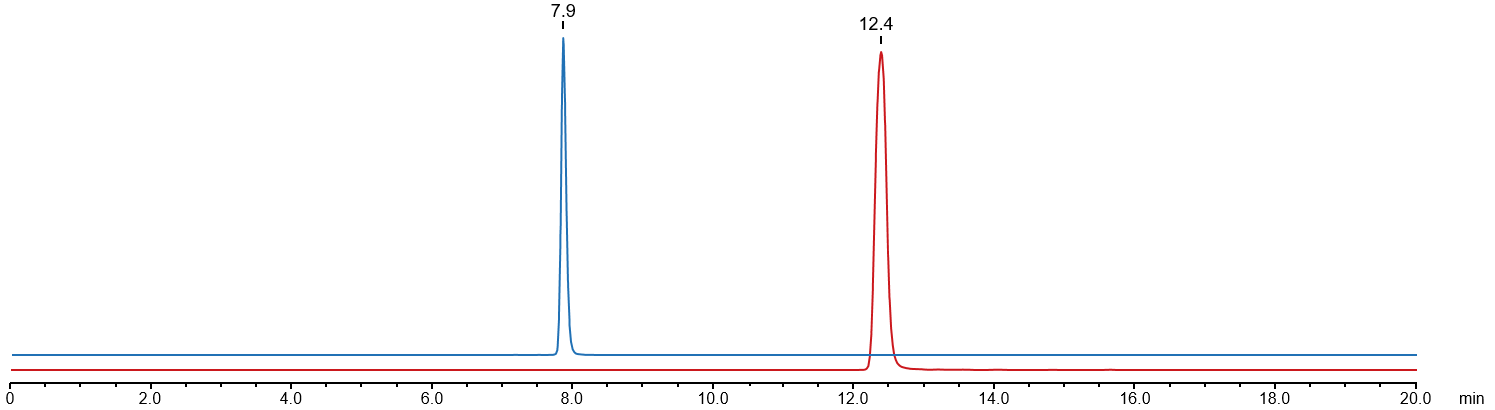


**S12.** Radio-HPLC chromatograms of Al[^18^F]F-**NOTA-6-Ahx-NonaLysan** (blue) and [^18^F]**SiFA-NonaLysan** (red). Column: Dr. Maisch ReproSil Pur C_18_ AQ, 150 x 4.6 mm, 5 µm, 120 Å; Solvent A: H_2_O/0.1 % TFA, Solvent B: MeCN/0.1 % TFA; Flow: 1 mL/min; Gradient: 5-60 % B in 15 min.


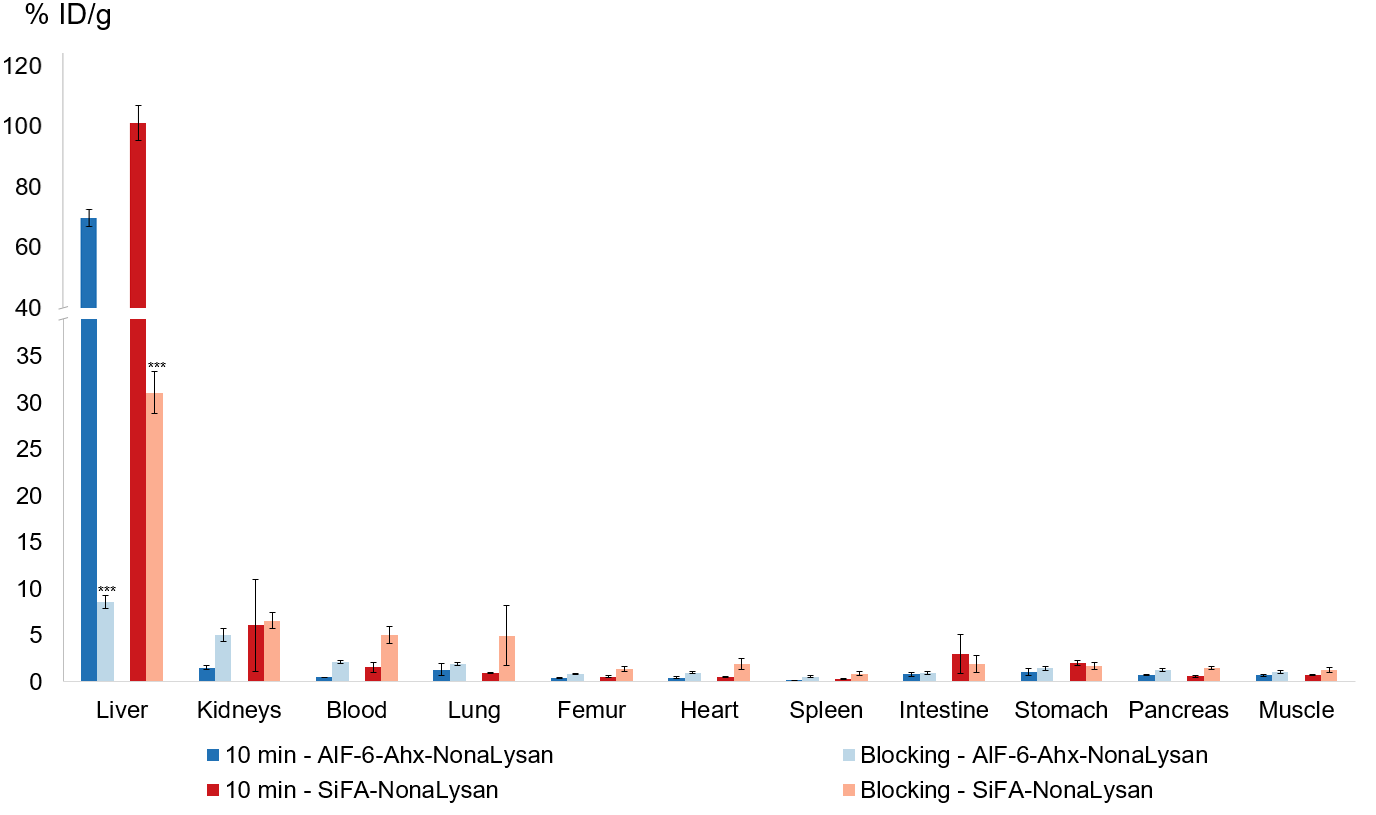


**S13.** Data (% ID/g) from the blocking experiments with Al[^18^F]F-**NOTA-6-Ahx-NonaLysan** & [^18^F]**SiFA-NonaLysan** in healthy BALB/c mice at 10 min p.i. Blocking was carried out by coinjecting 7.4 nmol of Galactosyl Serum Albumin (*** p ≤ 0.01).

**Table S14**. Biodistribution of Al[^18^F]F-**NOTA-6-Ahx-NonaLysan** (n = 3) & [^18^F]**SiFA-NonaLysan** *(*n = 3) in healthy BALB/c mice. Data are expressed as a percentage of the injected dose per gram (% ID/g), mean value ± standard deviation.

| Al[^18^F]F-**NOTA-6-Ahx-NonaLysan** | | | | | | [^18^F]**SiFA-NonaLysan** | | | | |
| --- | --- | --- | --- | --- | --- | --- | --- | --- | --- | --- |
|  | | | | | **blocking** |  | | | | **blocking** |
|  | 10 min | | 30 min | 60 min | 10 min | 10 min | 30 min | | 60 min | 10 min |
| **Liver** | 69.62 ± 2.76 | 76.18 ± 2.58 | | 47.93 ± 1.71 | 8.60 ± 0.68 | 101.19 ± 5.77 | | 20.85 ± 6.55 | 4.89 ± 3.61 | 31.06 ± 2.25 |
| **Kidneys** | 1.52 ± 0.23 | 1.32 ± 0.10 | | 1.33 ± 0.12 | 5.02 ± 0.65 | 6.06 ± 4.94 | | 1.32 ± 0.29 | 0.34 ± 0.02 | 6.58 ± 0.86 |
| **Blood** | 0.47 ± 0.02 | 0.41 ± 0.13 | | 0.39 ± 0.09 | 2.09 ± 0.19 | 1.55 ± 0.54 | | 0.58 ± 0.13 | 0.19 ± 0 | 5.00 ± 0.92 |
| **Lung** | 1.27 ± 0.65 | 0.95 ± 0.21 | | 0.77 ± 0.07 | 1.91 ± 0.20 | 0.92 ± 0.06 | | 0.72 ± 0.18 | 0.22 ± 0.03 | 4.94 ± 3.25 |
| **Femur** | 0.36 ± 0.03 | 0.46 ± 0.01 | | 0.22 ± 0.04 | 0.83 ± 0.10 | 0.54 ± 0.08 | | 1.09 ± 0.31 | 1.92 ± 0.08 | 1.36 ± 0.24 |
| **Heart** | 0.45 ± 0.07 | 0.42± 0.07 | | 0.40 ± 0.06 | 0.96 ± 0.13 | 0.49 ± 0.03 | | 0.32 ± 0.08 | 0.12 ± 0 | 1.87 ± 0.60 |
| **Spleen** | 0.14 ± 0.02 | 0.25 ± 0.08 | | 0.17 ± 0.05 | 0.51 ± 0.11 | 0.25 ± 0.05 | | 0.34 ± 0.28 | 0.11 ± 0.01 | 0.87 ± 0.19 |
| **Intestine** | 0.78 ± 0.20 | 7.81 ± 1.03 | | 15.98 ± 1.60 | 0.92 ± 0.13 | 2.98 ± 2.09 | | 48.34 ± 7.03 | 96.71 ± 32.14 | 1.89 ± 0.88 |
| **Stomach** | 1.05 ± 0.34 | 0.76 ± 0.18 | | 1.54 ± 0.20 | 1.43 ± 0.22 | 2.00 ± 0.27 | | 8.02 ± 8.26 | 7.87 ± 0.56 | 1.71 ± 0.40 |
| **Pancreas** | 0.72 ± 0.03 | 0.93 ± 0.08 | | 0.62 ± 0.04 | 1.27 ± 0.13 | 0.56 ± 0.07 | | 0.70 ± 0.30 | 0.15 ± 0.03 | 1.48 ± 0.16 |
| **Muscle** | 0.67 ± 0.09 | 0.70 ± 0.11 | | 0.47 ± 0.02 | 1.04 ± 0.17 | 0.70 ± 0.09 | | 0.36 ± 0.16 | 0.12 ± 0.02 | 1.29 ± 0.27 |
